# Genome-wide analysis of RNA-binding proteins co-expression with alternative splicing events in mitral valve prolapse

**DOI:** 10.3389/fimmu.2023.1078266

**Published:** 2023-04-26

**Authors:** Meng Zhao, Jingxin Zhou, Yihu Tang, Mingzhu Liu, Yawei Dai, Hui Xie, Zihao Wang, Liang Chen, Yanhu Wu

**Affiliations:** First Affiliated Hospital, Nanjing Medical University, Nanjing, Jiangsu, China

**Keywords:** mitral valve prolapse, RNA sequencing, RNA-binding protein, alternative splicing, genome-wide analysis

## Abstract

**Objectives:**

We investigated the role and molecular mechanisms of RNA-binding proteins (RBPs) and their regulated alternative splicing events (RASEs) in the pathogenesis of mitral valve prolapse (MVP).

**Methods:**

For RNA extraction, we obtained peripheral blood mononuclear cells (PBMCs) from five patients with MVP, with or without chordae tendineae rupture, and five healthy individuals. High-throughput sequencing was used for RNA sequencing (RNA-seq). Differentially expressed genes (DEGs) analysis, alternative splicing (AS) analysis, functional enrichment analysis, co-expression of RBPs, and alternative splicing events (ASEs) analysis were conducted.

**Results:**

The MVP patients exhibited 306 up-regulated genes and 198 down-regulated genes. All down- and up-regulated genes were enriched in both Gene Ontology (GO) terms and Kyoto Encyclopedia of Genes and Genomes (KEGG) pathways. Furthermore, MVP was closely associated with the top 10 enriched terms and pathways. In MVP patients, 2,288 RASEs were found to be significantly different, and four suitable RASEs (CARD11 A3ss, RBM5 ES, NCF1 A5SS, and DAXX A3ss) were tested. We identified 13 RNA-binding proteins (RBPs) from the DEGs and screened out four RBPs (ZFP36, HSPA1A, TRIM21, and P2RX7). We selected four RASEs based on the co-expression analyses of RBPs and RASEs, including exon skipping (ES) of DEDD2, alternative 3′ splice site (A3SS) of ETV6, mutually exclusive 3′UTRs (3pMXE) of TNFAIP8L2, and A3SS of HLA-B. Furthermore, the selected four RBPs and four RASEs were validated by reverse transcription–quantitative polymerase chain reaction (RT-qPCR) and showed high consistency with RNA sequencing (RNA-seq).

**Conclusion:**

Dysregulated RBPs and their associated RASEs may play regulatory roles in MVP development and may therefore be used as therapeutic targets in the future.

## Introduction

Degenerative mitral valve disease is one of the most common degenerative heart diseases. This disease has become one of the leading causes of cardiovascular morbidity and mortality in developed nations, with morbidity ranging from 2% to 3% (1). Degenerative mitral valve disease is characterized by lengthening or rupture of the chordae tendineae, resulting in mitral valve prolapse (MVP). Most individuals with MVP have chest tightness, and investigations have shown that this disease is associated with catastrophic outcomes such as heart failure, atrial fibrillation, arterial thrombosis, lethal ventricular arrhythmia, and even sudden cardiac death (2).

Mitral valve repair has grown increasingly popular in recent years, as it improves left ventricle (LV) function (1). However, despite the decreased surgical mortality (3) and improved long-term survival rate (4) associated with mitral valve repair, it has been found that approximately one-third of patients who receive mitral valve surgery develop moderate to severe mitral regurgitation (5). Icardo (6) discovered that the normal chordae of MVP patients exhibited pathological alterations in their microstructures. In addition, mitral valve repair cannot halt the progression of MVP (7), and therefore some researchers believe that mitral valve replacement may be required (6). However, the limitations of mitral valve replacement, which include the short lifespan of the bioprosthetic valve and anticoagulation of the mechanical valve, make patients, particularly young people, reluctant to undergo this procedure As a result, understanding the etiology of MVP is critical. Very little research on the mechanisms of MVP’ has been carried out.

RNA-binding proteins (RBPs), by binding to target RNA *via* an RNA recognition domain (8), can regulate the alternative splicing (AS) of pre-mRNA, and they also contain domains that enable them to bind with other proteins, allowing RBPs to perform their regulatory functions (9). RBPs and mRNA alternative splicing events (ASEs) have been identified as effectors and regulators in cardiovascular disorders, respectively (10). However, no data on the relationship between RBPs and MVPs have been documented yet.

As a result, in this study, high-throughput sequencing was employed for RNA sequencing (RNA-seq) in MVP patients, and genome-wide analysis of the co-expression of RBPs and ASEs was performed to evaluate the potential pathogenic components in the circulatory systems of MVP patients.

## Materials and methods

### Human blood

Patients with MVP who were admitted to Jiangsu Provincial Hospital for mitral valve surgery between February and August 2022 were chosen, and comparably healthy examinees were selected as the control group. The following were the inclusion criteria for the study group (1): the patient was diagnosed with MVP with or without chordae tendineae rupture by echocardiography and had no severe aortic or tricuspid valve dysfunction (2); the patient had moderate to severe mitral regurgitation and required surgical intervention; and (3) coronary angiography ruled out coronary heart disease. The control group’s inclusion criterion was that echocardiography was used to exclude cardiac valve disorders. The patient’s blood was drawn shortly after admission to the hospital, and peripheral blood mononuclear cells (PBMCs) were taken for RNA extraction.

### RNA extraction and sequencing

Trizol was used to extract total RNA (Ambion, 15596-018). Total RNA was treated with RQ1 DNase (Promega) to remove DNA. A SmartSpec Plus™ spectrophotometer (Bio-Rad) was used to determine the quality and quantity of RNA by measuring the absorbance at 260 nm/280 nm (A_260_/A_280_). Electrophoresis with a 1.5% agarose gel was used to confirm RNA integrity.

RNA-seq libraries were created for each sample using 1 μg of total RNA. mRNAs were captured using VAHTS mRNA capture Beads (Vazyme, N401). The purified RNA was treated with RQ1 DNase (Promega) for the removal of DNA before being utilized for the directional RNA-seq library created using the KAPA Stranded mRNA-Seq Kit for Illumina^®^ Platforms (KK8544). Polyadenylated mRNAs were purified and fragmented. Fragmented mRNAs were then converted into double-stranded cDNA. Following end repair and A tailing, the DNAs were ligated to a Diluted Roche Adaptor (KK8726). After purification of the ligation product and size fractioning to 300–500 bp, the ligated products were amplified, purified, quantified, and stored at –80°C before sequencing. The strand marked with dUTP (i.e., the second cDNA strand) is not usually amplified, allowing for strand-specific sequencing.

The manufacturer’s instructions were followed for high-throughput sequencing and an Illumina NovaSeq 6000 system was used for 150 nt paired-end sequencing of the cDNA libraries.

### RNA-seq raw data clean and alignment

Raw reads containing more than 2N bases were initially discarded. A FASTX-Toolkit (version 0.0.13) was used to trim adaptors and low-quality bases. Short reads of fewer than 16 nt were discarded. Subsequently, clean reads were aligned to the GRCh38 genome using HISAT2 (11), with four mismatches allowed. Uniquely mapped reads were used to quantify the numbers of gene reads and calculate the fragments per kilobase of transcript per million fragments mapped (FPKM) (12).

### Analysis of differentially expressed genes

Differentially expressed genes (DEGs) were identified using the R Bioconductor program DESeq2 (13). The cut-off parameters for detecting DEGs were fold change (FC) > 1.5 or < 0.67 and a *p*-value of < 0.01.

### AS analysis

The ASEs and regulated ASEs between the samples were identified and quantified by using the ABLas pipeline in accordance with the method described in previous studies (14, 15). In brief, ABLas detection of 10 types of ASEs was based on the splice junction reads, including exon skipping (ES), alternative 5′ splice site (A5SS), alternative 3′ splice site (A3SS), mutually exclusive exons (MXEs), mutually exclusive 5′UTRs (5pMXE), mutually exclusive 3′UTRs (3pMXE), cassette exon, A3SS&ES, and A5SS&ES.

### Functional enrichment analysis

A KOBAS 2.0 server (16) was used to identify Gene Ontology (GO) terms and Kyoto Encyclopedia of Genes and Genomes (KEGG) pathways so that DEGs could be categorized into functional groups. To determine the enrichment of each term, a hypergeometric test and the Benjamini–Hochberg false discovery rate (FDR) controlling process were utilized.

### Co-expression analysis

Co-expression analysis was performed for every differentially expressed RBP (DERBP) and regulated alternative splicing event (RASE). The Pearson correlation coefficient between DERBP and RASE was determined, and DERBP–RASE relationship pairs achieving an absolute correlation coefficient of ≥ 0.8 and *p*-value ≤ 0.01 were screened.

### Protein–protein interaction network construction analysis

The Search Tool for the Retrieval of Interacting Genes (STRING) (http://www.string-db.org) was used to investigate the relationship between DEGs and RBPs. The minimum required interaction score was set to 0.4, and protein nodes that had no interaction with others were eliminated.

### Validation of important RBPs and regulated alternative splicing genes in clinical samples

The glyceraldehyde-3-phosphate dehydrogenase (GAPDH) gene was used as a control gene to analyze the relative expression of specific genes. Standard cDNA synthesis techniques were followed, and RT-qPCR was performed on a Bio-Rad S1000 with Hieff qPCR SYBR^®^ Green Master Mix (Low Rox Plus; YEASEN, China). Primer data are available in **Supplementary File 1**. The concentration of each transcript was then normalized to GAPDH mRNA level using the 2^–ΔΔCT^ method (17).

A list of the primers that were used for the detection of pre-mRNA splicing, and subsequently for the quantitative evaluation of the two distinct splicing isoforms of a given ASE using the quantitative polymerase chain reaction (qPCR) technique, is given in **Supplementary File 1**. To precisely amplify each of these two isoforms, primers complementary to the splice junction of the constitutive exon and alternative exon were designed. Standard cDNA synthesis protocols were followed, and RT-qPCR was conducted using a Bio-Rad S1000 with Hieff qPCR SYBR^®^ Green Master Mix (Low Rox Plus; YEASEN, China). The 2^–ΔΔCT^ technique was used to quantify PCR amplifications.

### Other statistical analysis

Principal component analysis (PCA) was performed using the R package factoextra (https://cloud.r-project.org/package=factoextra/) to illustrate the clustering of data with the first two components. After normalizing the reads in samples by tags per million (TPM), in-house software (Sogen) was used to visualize next-generation sequence data and genomic annotations. In addition, the pheatmap package (https://cran.r-project.org/web/packages/pheatmap/index.html/) in R was used to perform the clustering based on Euclidean distance. For comparisons between the two groups, a Student’s *t*-test and a Fisher’s precision probability test were employed, and the results were presented as means ± standard deviation (SD).

### Availability of data and materials

The raw data supporting the conclusion of this article will be made available by the authors, without undue reservation.

## Results

### Quality control of sequencing data

The sequencing data were subjected to quality control to exclude low-quality bases and reads, and therefore the sequences that were preserved were of high quality. The project’s clean ratio was greater than 97%, while the basic quality of clean reads (Q20) was greater than 97% (*p* < 0.01). Q30 (*p* < 0.001) was greater than 93%. (**Table S1**). Q20 and Q30 are the probabilities of error detection of bases; Q20 is 0.01.

### DEGs in the MVP patient and healthy individual groups

This study enrolled five MVP patients and five age- and gender-matched healthy individuals. All MVP patients had significant mitral regurgitation and required mitral valve surgery, but none of them developed heart failure. One MVP patient had undergone a cholecystectomy 1 year prior. **Table 1** compares the characteristics of the two groups. Blood was drawn from 10 participants, and PBMCs were taken for RNA extraction.

**Table 1:** 

P2RX7-F	GAGCCTGTCATCAGTTCTG
P2RX7-R	TGTAGTCTGCGGTGTCAA
ZFP36-F	CTGTCTCCTAGAATCTTATGTG
ZFP36-R	GCTTTGGCTACTTGCTTT
TRIM21-F	TATAAGGAGGCTGCTTCAC
TRIM21-R	ATGAACTCTGAACCACCTT
HSPA1A-F	CAGTTCTCAATTTCCTGTGT
HSPA1A-R	TAGTCGTAAGATGGCAGTAT
ETV6-M/AS-F	TTTACTGGAGCAGGGATGAC
ETV6-AS-R	GAGTCGAGGTCTGAATGAGG
ETV6-M-R	GCACATCACCTGAATGAGG
DEDD2-M/AS-F	GCTACCCTCTGTCCTCTTTGA
DEDD2-M-R	GCCGGCCAGTGTCTCCAGAA
DEDD2-AS-R	CAGAATCTGTGTCTCCAGAA
HLA-B-M-F	CCTTTTCCACCCCATCTCAG
HLA-B-AS-F	GGGAAGACGGCCCATCTCAG
HLA-B-M/AS-R	CTGTGGTGGTGCCTTCTGGA
TNFAIP8L2-M/AS-F	GCTTCTCCATTCTGTAAGCT
TNFAIP8L2-M-R	TGGTCAGTCACTGCTGTGCT
TNFAIP8L2-AS-R	CTCTTCTTTTCTGCTGTGCT

hum-GAPDH-F: GGTCGGAGTCAACGGATTTG.

hum-GAPDH-R: GGAAGATGGTGATGGGATTTC.

**Figure 1:**
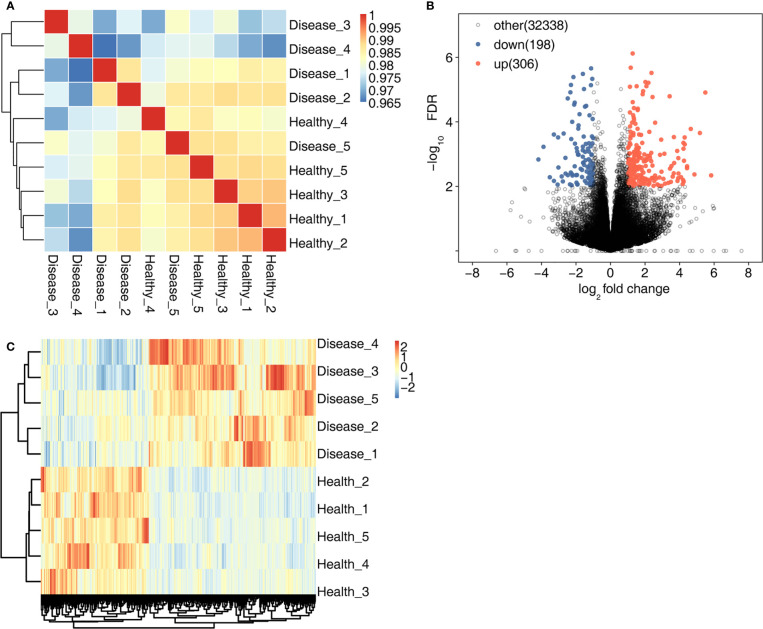
DEGs in the MVP patient and healthy individual groups. **(A)** shows an inter-sample clustering analysis of sample correlation. The 306 up-regulated genes and 198 down-regulated genes are shown in **(B)** A heat map of 504 DEGs is shown in **(C)** DEGs, differentially expressed genes; MVP, mitral valve prolapse.

High-throughput sequencing was used for RNA-seq. First, sample correlation analysis was performed to evaluate the transcript expression values for all samples and to reveal the comparability of each sample (**Figure 1A**). A total of 504 DEGs were detected in the MVP patient and healthy individual groups. Compared with the healthy individual group, the MVP patient group exhibited 306 up-regulated genes and 198 down-regulated genes. All DEGs identified in the samples of MVP patients and healthy individuals are shown in the form of a volcano plot (**Figure 1B**) and a hierarchical clustering heat map (**Figure 1C**). All DEGs and RASEs are listed in **Tables 4**, **5**, **6** In The **Supplementary Files**.

### Functional enrichment analysis of DEGs between MVP group and healthy group

**Figure 2:**
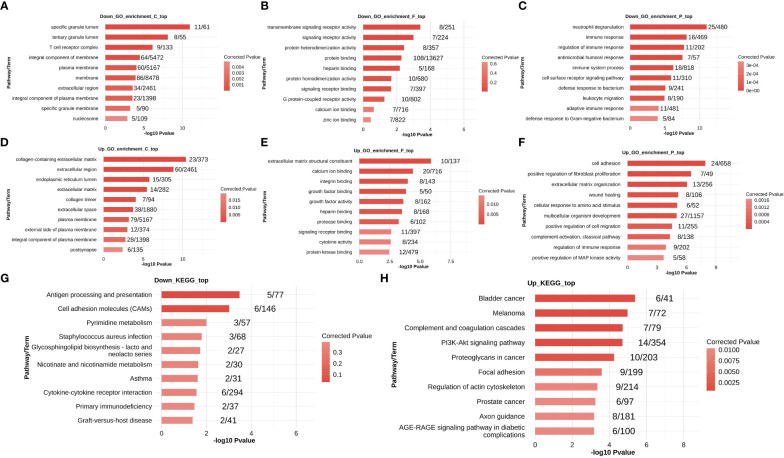
Functional enrichment analysis of DEGs in the MVP patient and healthy individual groups. The down-regulated DEGs that were enriched in GO terms are shown in panels **(A–C)**, and up-regulated DEGs that were enriched in GO terms are shown in panels **(D–F)**. The down- and up-regulated DEGs that were enriched in KEGG pathways are shown in **(G, H)**, respectively. DEG, differentially expressed gene; GO, Gene Ontology; MVP, mitral valve prolapse.

Gene Ontology terms consist of cellular components, molecular functions, and biological processes. The down- and up-regulated DEGs were enriched in the three components of GO terms, and the top 10 most enriched terms are shown in **Figures 2A–F**. The down-regulated DEGs were remarkably enriched in the terms neutrophil degranulation, immune response, and immune response regulation, whereas up-regulated DEGs were enriched in the terms cell adhesion, positive regulation of fibroblast proliferation, extracellular matrix organization, wound healing, extracellular matrix structural constituents, and collagen-containing extracellular matrix.

The top 10 most enriched KEGG pathways are shown in **Figures 2G, H**. The down-regulated DEGs were largely abundant in antigen processing and presentation and cell adhesion molecules, whereas up-regulated DEGs were associated with the terms bladder cancer, melanoma, complement and coagulation cascades, and PI3K-Akt signaling pathways.

### The AS analysis of peripheral blood in MVP patients

**Figure 3:**
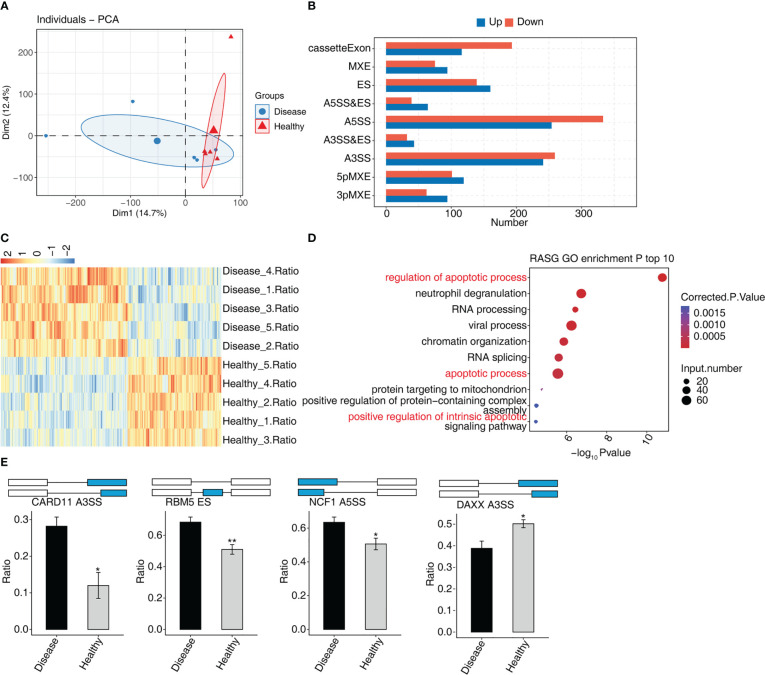
The AS analysis of peripheral blood in MVP patients. **(A)** Principal component analysis of two sample groups of samples based on normalized NIR splicing ratio. **(B)** The bar chart shows the distribution of AS events in nine AS types. X-axis: the different types of AS events. Y-axis: RASE number. **(C)** Hierarchical clustering heat map showing expression levels of all RASEs. **(D)** Bubble diagram exhibiting the most enriched GO biological process results of the regulated alternative splicing genes (RASGs). **(E)** Bar plot showing the expression pattern and statistical difference of RASE. AS, alternative splicing; GO, Gene Ontology; MVP, mitral valve prolapse; RASE, regulated alternative splicing event. * means p<0.05, ** means p<0.01.

In MVP patients, 2,288 RASEs were shown to be significantly different, with 995 RASEs being down-regulated and 1,293 being up-regulated. PCA of two groups of samples based on normalized non-intron retention regulated (NIR) splicing ratio revealed a high degree of similarity between the two groups (**Figure 3A**). The distribution of AS events in nine AS types indicated that the RASEs were concentrated mainly on A5SS, A3SS, and cassette exons (**Figure 3B**). The expression levels of all RASEs were displayed on a hierarchical clustering heat map, as shown in **Figure 3C**. All regulated alternative splicing genes (RASGs) were enriched in biological process GO terms (**Figure 3D**). The apoptosis-related terms were among the top 10 enriched terms. As a result, four suitable RASEs in the apoptosis-related terms were screened based on their *p*-values: CARD11 A3ss, RBM5 ES, NCF1 A5SS, and DAXX A3ss. CARD11 A3ss, RBM5 ES, and NCF1 A5SS were significantly expressed in the MVP group, whereas DAXX A3ss was low in the MVP group compared with the healthy group (**Figure 3E**).

### Analysis of differential expression of RBPs and co-expression network between RBPs and RASGs associated MVP patients

**Figure 4:**
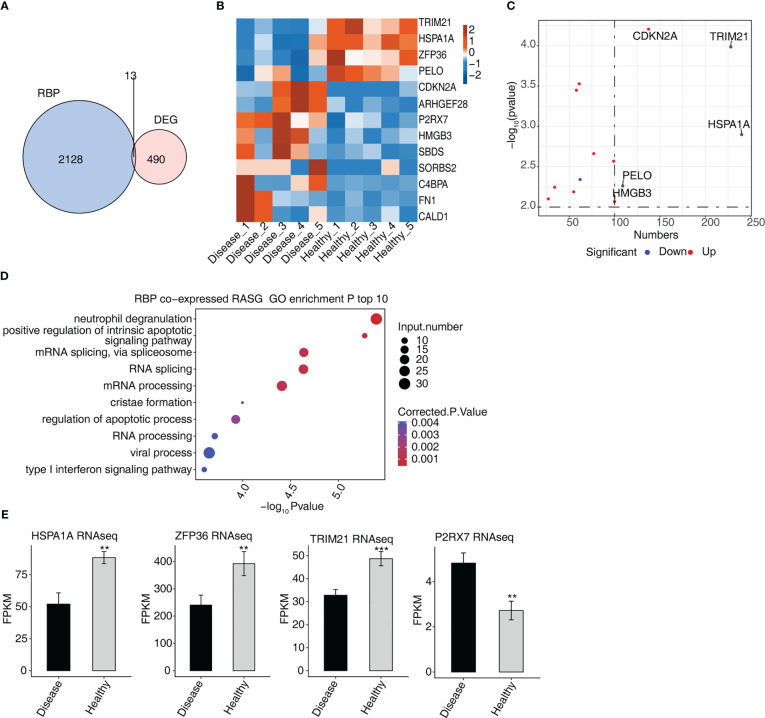
Analysis of differential expression of RBPs in MVP patients **(A)** Venn diagram showing the overlap in the number of genes of RBPs and DEGs. **(B)** Hierarchical clustering heat map showing the expression levels of overlapping RBPs. **(C)** The scatterplot shows the RBPs and the number of co-expressed RASGs. **(D)** Bubble diagram exhibiting the most enriched GO biological process results of the RBPs co-expressed RASGs. **(E)** Bar plot showing the expression pattern and statistical difference of RBPs. DEG, differentially expressed gene; GO, Gene Ontology; MVP, mitral valve prolapse; RASG, regulated alternative splicing gene; RBP, RNA-binding protein. ** means p<0.01, *** means p<0.001.

Thirteen genes associated with DEGs and RBPs were found to intersect, i.e., were found in both groups, of which four were down-regulated and nine were up-regulated (**Figure 4A**). In a hierarchical clustering heat map, the expression levels of 13 RBPs were compared between the two groups (**Figure 4B**). ZFP36 interacted with SBDS, FN1, CDKN2 A, CALD1, and C4BPA in a protein–protein interaction (PPI) comprising 13 RBPs (**Figure S1A**).

The network diagram of co-expressed RASGs and RBPs revealed that 13 RBPs and 1,291 RASEs were co-expressed (**Figure S1B**). The 13 RBPs and the number of co-expressed RASGs are shown in **Figure 4C**. Co-expressed RASGs and RBPs were enriched in GO biological process terms, and the most enriched terms were neutrophil degranulation, positive regulation of intrinsic apoptotic signaling pathway, mRNA splicing *via* spliceosome, RNA splicing, mRNA processing, cristae formation, regulation of the apoptotic process, RNA processing, viral process, and type I interferon signaling pathway (**Figure 4D**). Following a literature review, we chose four RBPs (HSPA1A, ZFP36, TRIM21, and P2RX7) that play important roles in the development of cardiovascular diseases (18–26) for further co-expression analysis (**Figure 4E**).

**Figure 5:**
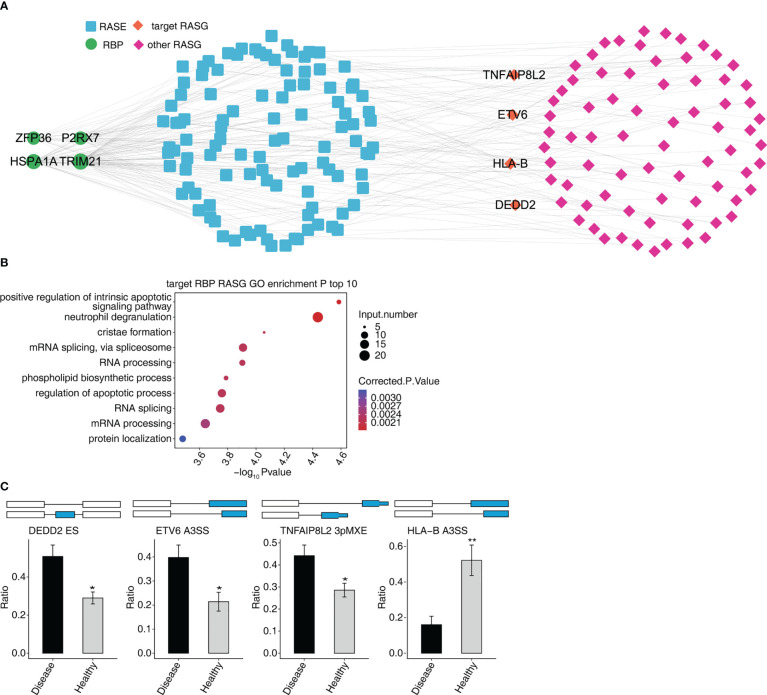
Co-expression network between DE RBPs and RASGs associated MVP patients. **(A)** The network diagram shows co-expression between RASGs with ZFP36, HSPA1A, TRIM21, and P2RX7. **(B)** Bubble diagram exhibiting the most enriched GO biological process results of the four RBPs co-expressed with RASGs. **(C)** Bar plot showing the expression pattern and statistical difference of RASEs. DEG, differentially expressed gene; GO, Gene Ontology; MVP, mitral valve prolapse; RASG, regulated alternative splicing gene; RBP, RNA-binding protein; DE RBPs, differentially expressed RBPs. * means p<0.05, ** means p<0.01.

The four RBPs (HSPA1A, ZFP36, TRIM21, and P2RX7) co-expressed with 629 RASEs, and 276 RASGs. We selected four RASEs (DEDD2 ES, ETV6 A3SS, TNFAIP8L2 3pMEX, and HLA-B A3SS) among 629 RASEs based on their expression levels, the significance of difference (the *p*-value), and the literature study (**Figure 5C**) (27–30). **Figure 5** shows the co-expression of four RASGs (DEDD2, ETV6, TNFAIP8L2, and HLA-B) with four RBPs (HSPA1A, ZFP36, TRIM21, and P2RX7) as well the highest enriched GO biological process terms. [Positive regulation of intrinsic apoptotic signaling pathway, neutrophil degranulation, cristae formation, mRNA splicing *via* spliceosome, RNA processing, phospholipid biosynthetic process, regulation of the apoptotic process, RNA splicing, mRNA processing, and protein localization (**Figure 5B**)].

### Validation of clinically important RBPs and RASGs

*P2RX7* gene expression was dramatically increased in the MVP patient group compared with the control group, whereas *ZFP36*, *TRIM21*, and *HSPA1A* gene expressions were significantly decreased.

**Figure 6:**
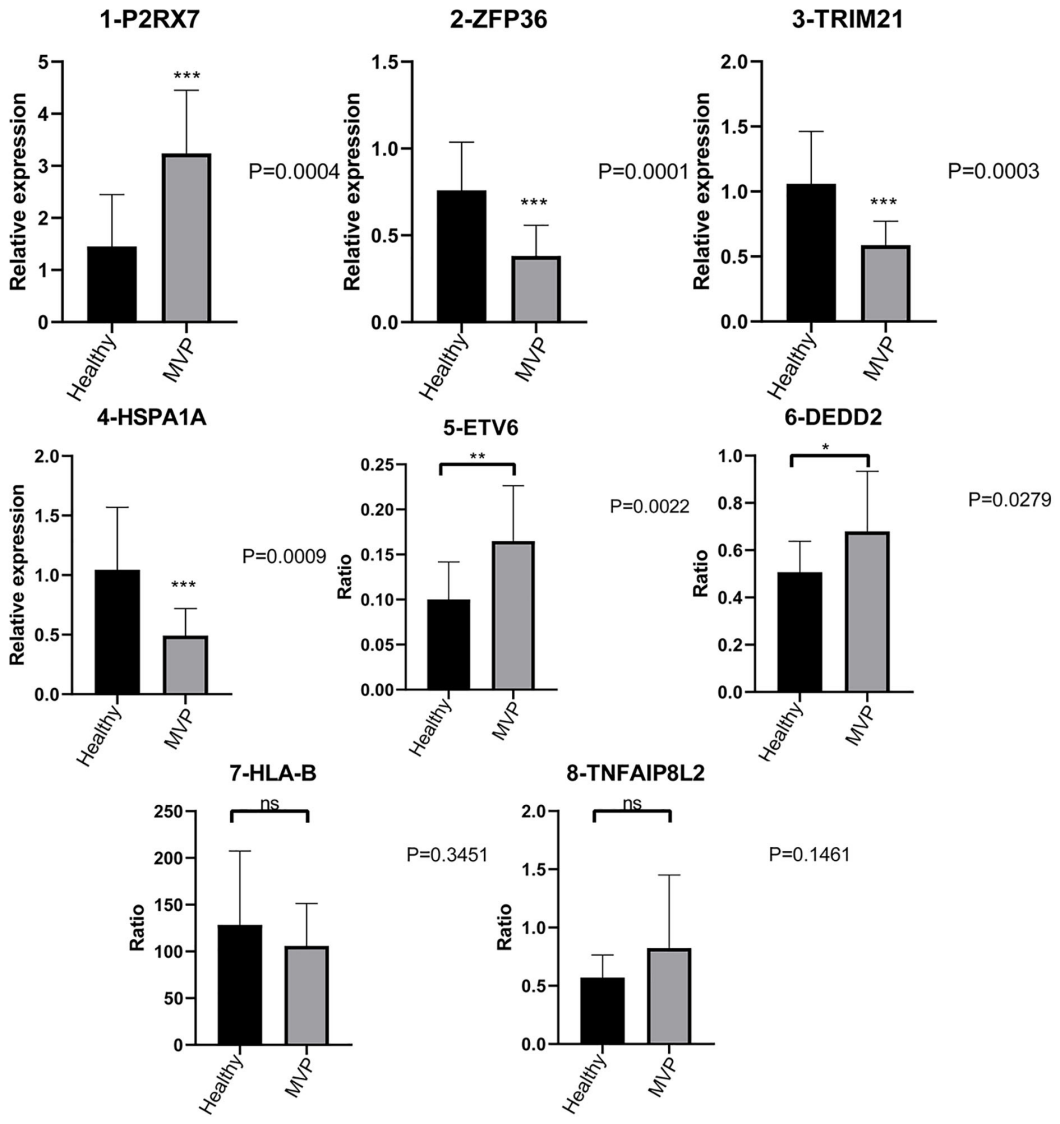
Validation of important RBPs and RASGs in clinical samples. Results of the qPCR of the gene expression of four RPBs and four AS events of RASGs. AS, alternative splicing; RAS, regulated alternative splicing gene; RBP, RNA-binding protein; qPCR, quantitative polymerase chain reaction.

Furthermore, there were considerably more ASEs in the *DEDD2* and *ETV6* genes in the MVP patient group than in the group of healthy individuals; however, the numbers of ASEs in the *HLA-B* and *TNFAIP8L2* genes were not significantly different between the two groups (**Figure 6**).

## Discussion

Because of the use of 3D echocardiography and cardiac magnetic resonance imaging (MRI), a consensus in the areas of diagnosis and therapy of MVP has been reached. However, questions related to the genetics and mechanisms of MVP remain unresolved. Consequently, we used RNA-seq to investigate the probable pathogenesis in the MVP circulatory system. We identified four suitable RBPs and four RASGs that could be implicated in the development of MVP after thoroughly analyzing the RNA-seq results.

Transforming growth factor β (TGF-β) was the most clearly identified pathogenic factor for MVP in previous studies (31–33). It can stimulate collagen secretion and result in the remodeling of the extracellular matrix (ECM) (33). In our study, we found that the most enriched GO terms of DEGs were extracellular matrix organization, extracellular matrix structural constituent, and collagen-containing extracellular matrix. This agrees with previous findings. Thus, changes in the ECM were the key manifestation of MVP. Further investigation is needed to determine which factors cause ECM modifications and how these changes contribute to MVP. In addition, as shown in **Figure 2C**, neutrophil degranulation was the most enriched term. In our study, we enriched 25 DEGs into neutrophil degranulation terms. Among these 25 DEGS, *CXCR1* has been shown to mediate neutrophil degranulation in an *in vivo* experiment (34). It was also found to be a surface marker of monocytes (35) in cardiovascular diseases. Thus, neutrophil degranulation may play an important role in MVP progression.

A previous study linked *FBN1*, *PKD1*, *DCHS1*, and *DZIP1* to MVP (1). However, these genes were all linked to familial MVP or MVP as part of a syndrome of connective tissue disorders (CTDs) such as Marfan syndrome, adult polycystic kidney disease, or Ehlers–Danlos syndrome (1). Our study focused on sporadic MVP, and our results indicated that the expression levels of these four genes did not change significantly.

TGF-β plays an integral role in regulating immune responses (36) and has intricate relationships with the immune system (37). In our study, DEGs were also significantly enriched in the immune response terms in GO enrichment analysis, and the four RBPs identified were all associated with immune-inflammatory responses.

ZFP36 is a well-known anti-inflammatory modulator that can both reduce the production of pro-inflammatory cytokines and control various immune responses (38). ZFP36 has been linked to a variety of autoimmune diseases such as rheumatoid arthritis, psoriasis, multiple sclerosis, and juvenile idiopathic arthritis (39). The P2X7 receptor (P2RX7) is mainly expressed in immune cells and serves as an essential regulator of inflammation, immunity, and cellular death (21, 40). P2RX7 is associated with several cardiovascular diseases, including hypertension, atherosclerosis, ischemia/reperfusion injury, and heart failure (21). This is due to its role in promoting endothelial dysfunction and inflammation. HSPA1A is a heat shock protein with anti-apoptotic and antithrombotic properties. It has been shown that HSPA1A can protect against atherosclerosis because of its anti-inflammatory and antithrombotic characteristics (41). *TRIM21* is critical to defense against invading viruses (42). A study by Bolland (43) found that a lack of Ro52/Trim21 can exacerbate injury-induced systemic autoimmune illness *via* the IL-23–Th17 pathway. Anti-*TRIM21* antibodies have also been identified in a variety of autoimmune disorders (44, 45).

In summary, three RBPs (ZFP36, HSPA1A, and TRIM21) with anti-inflammatory functions were decreased in the MVP patient group, whereas P2RX7, which can enhance the inflammatory response, was increased in the MVP patient group compared with the healthy individual group. We, therefore, concluded that immune-inflammatory responses may play a crucial role in the development and progression of MVP. Further research is needed to determine if the four RBPs tested are linked with TGF-β and how they contribute to the advancement of MVP.

In addition, HSPA1A has been linked to a lower incidence of pulmonary fibrosis (46). Research has found that fibrosis and matrix remodeling of the mitral valve are major manifestations of MVP (32). Thus, HSPA1A may protect against MVP by decreasing the deposition of collagen in the ECM of the mitral valve.

The complexity of the proteome is increased by alternative pre-mRNA splicing. A dysfunctional splicing process has been identified in several diseases (47), which may suggest possible treatment targets for these diseases (48). Previous research has linked AS to atherosclerosis, heart failure, and dilatative cardiomyopathy (49). However, no studies have found a link between the AS of mRNA and MVP.

We discovered significant changes in the ASEs of four RASGs (DEDD2, ETV6, TNFAIP8L2, and HLA-B). The ASEs of the *DEDD2*, *ETV6*, and *TNFAIP8L2* genes were considerably increased in the MVP group and were associated with apoptosis (28, 50–52). HLA-B ASEs, which inhibited apoptosis and cell invasion while reducing cell proliferation (53), were significantly reduced in the MVP group. Furthermore, three of the top 10 most enriched terms in the RASGs GO enrichment analysis (**Figure 3D**) were associated with apoptosis.

Until now, few studies have focused on the apoptosis of mitral valve cells in MVP. Thus, our findings provided a breakthrough point for the study of MVP mechanism.

### Limitations

There are several drawbacks to this study. Firstly, the sample size of this study is quite small and therefore larger sample size studies ought to be conducted in future. Secondly, since the sample for this study was peripheral blood, the RNA-seq results may have been influenced by other factors, such as hypertension, diabetes, and heart function. Thirdly, because this study only used clinical samples, *in vitro* and animal investigations are required to validate the pathogenicities of the four RBPs and four RASGs to MVP.

## Conclusion

Immune-inflammatory responses may contribute to the development and progression of MVP by accelerating the apoptosis of mitral valve cells. ZFP36, HSPA1A, TRIM21, and P2RX7 may be involved in the regulation of the AS of *DEDD2*, *ETV6*, *TNFAIP8L2*, and *HLA-B*, and hence play an important role in the development of MVP.

## Data availability statement

The datasets presented in this study can be found in online repositories. The names of the repository/repositories and accession number(s) can be found below:GSE229778 (GEO).

## Ethics statement

The studies involving human participants were reviewed and approved by the Ethics Committee of Jiangsu Provincial People’s Hospital. The patients/participants provided their written informed consent to participate in this study.

## Author contributions

YW and LC contributed to the conception and design of the study. MZ, JZ, and YT performed the molecular analysis. MZ and JZ performed bioinformatics analysis. YD, ML, HX, and ZW collected and processed the human blood. MZ, YD, and HX wrote the first draft of the manuscript. All authors contributed to the article and approved the submitted version.

## Supplementary material

Supplementary Figure 1 The results of AS analysis of blood samples from patients with MVP. **(A)** PPI diagrams of RBPs in STRING. **(B)** The network diagram shows the co-expressed RASGs with 13 RBPs. AS, alternative splicing; MVP, mitral valve prolapse; PPI, protein–protein interaction; RASG, regulated alternative splicing gene; RBP, RNA-binding protein; STRING, Search Tool for the Retrieval of Interacting Genes.
