# Supplementary figures and images for: Genome-wide analysis of RNA-binding proteins co-expression with alternative splicing events in mitral valve prolapse
Source: Front Immunol. 2023 Apr 26;14:1078266. doi: 10.3389/fimmu.2023.1078266 (PMC10171460; doi:10.3389/fimmu.2023.1078266)

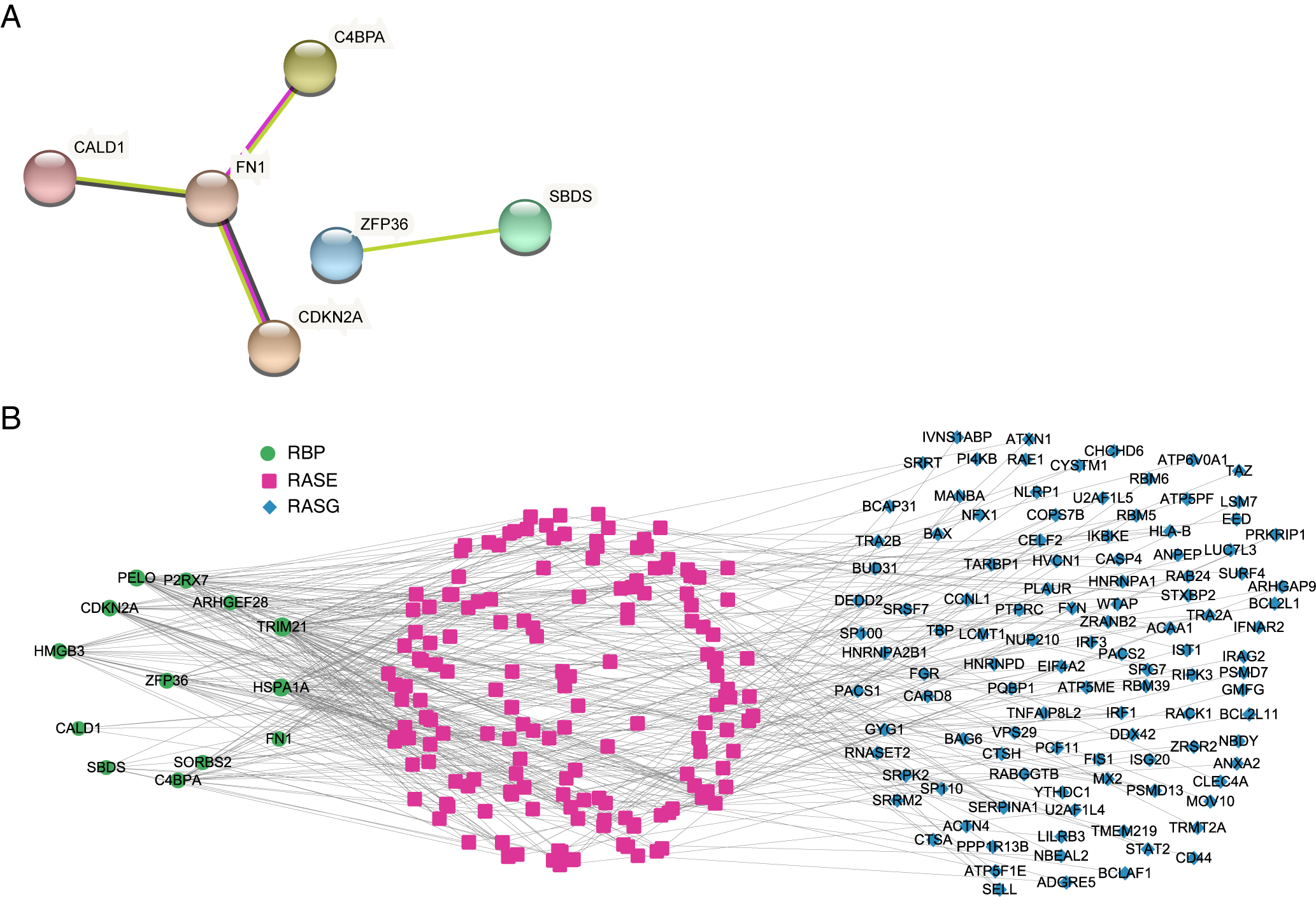

Supplement: Supplementary Figure 1 — The results of AS analysis of blood samples from patients with MVP. (A) PPI diagrams of RBPs in STRING. (B) The network diagram shows the co-expressed RASGs with 13 RBPs. AS, alternative splicing; MVP, mitral valve prolapse; PPI, protein–protein interaction; RASG, regulated alternative splicing gene; RBP, RNA-binding protein; STRING, Search Tool for the Retrieval of Interacting Genes. [file Image_1.tif]
