# Supplementary material for: Genome-wide analysis of RNA-binding proteins co-expression with alternative splicing events in mitral valve prolapse
Source: Front Immunol. 2023 Apr 26;14:1078266. doi: 10.3389/fimmu.2023.1078266 (PMC10171460; doi:10.3389/fimmu.2023.1078266)
Supplement: Supplementary file 2 [file Table_1.docx]

hum-GAPDH-F： GGTCGGAGTCAACGGATTTG

hum-GAPDH-R： GGAAGATGGTGATGGGATTTC

| P2RX7-F | GAGCCTGTCATCAGTTCTG |
| --- | --- |
| P2RX7-R | TGTAGTCTGCGGTGTCAA |
| ZFP36-F | CTGTCTCCTAGAATCTTATGTG |
| ZFP36-R | GCTTTGGCTACTTGCTTT |
| TRIM21-F | TATAAGGAGGCTGCTTCAC |
| TRIM21-R | ATGAACTCTGAACCACCTT |
| HSPA1A-F | CAGTTCTCAATTTCCTGTGT |
| HSPA1A-R | TAGTCGTAAGATGGCAGTAT |
| ETV6-M/AS-F | TTTACTGGAGCAGGGATGAC |
| ETV6-AS-R | GAGTCGAGGTCTGAATGAGG |
| ETV6-M-R | GCACATCACCTGAATGAGG |
| DEDD2-M/AS-F | GCTACCCTCTGTCCTCTTTGA |
| DEDD2-M-R | GCCGGCCAGTGTCTCCAGAA |
| DEDD2-AS-R | CAGAATCTGTGTCTCCAGAA |
| HLA-B-M-F | CCTTTTCCACCCCATCTCAG |
| HLA-B-AS-F | GGGAAGACGGCCCATCTCAG |
| HLA-B-M/AS-R | CTGTGGTGGTGCCTTCTGGA |
| TNFAIP8L2-M/AS-F | GCTTCTCCATTCTGTAAGCT |
| TNFAIP8L2-M-R | TGGTCAGTCACTGCTGTGCT |
| TNFAIP8L2-AS-R | CTCTTCTTTTCTGCTGTGCT |
