# Supplementary material for: Genome-wide analysis of RNA-binding proteins co-expression with alternative splicing events in mitral valve prolapse
Source: Front Immunol. 2023 Apr 26;14:1078266. doi: 10.3389/fimmu.2023.1078266 (PMC10171460; doi:10.3389/fimmu.2023.1078266)
Supplement: Supplementary file 3 [file Table_2.docx]

**Obtain the high-quality clean reads**

| Sample | raw(total) | clean(total) | ratio(total) | raw(base) | clean(base) | ratio(base) | Q20 | Q30 | GC | DUP |
| --- | --- | --- | --- | --- | --- | --- | --- | --- | --- | --- |
| Healthy 1 | 90451552 | 88513393 | 97.86% | 13.568G | 11.960G | 88.15% | 97.89 | 93.69 | 46% | 71.10% |
| Healthy 2 | 83734256 | 81961981 | 97.88% | 12.560G | 10.998G | 87.56% | 97.87 | 93.66 | 46% | 70.25% |
| Healthy 3 | 89027096 | 87035009 | 97.76% | 13.354G | 11.841G | 88.67% | 97.86 | 93.64 | 46% | 71.40% |
| Healthy 4 | 78073410 | 75975692 | 97.31% | 11.711G | 10.204G | 87.13% | 97.79 | 93.56 | 48% | 73.70% |
| Healthy 5 | 80996768 | 79065557 | 97.62% | 12.150G | 10.746G | 88.45% | 97.87 | 93.72 | 47% | 72.42% |
| Disease 1 | 80882214 | 79147058 | 97.85% | 12.132G | 10.704G | 88.22% | 97.91 | 93.74 | 47% | 69.12% |
| Disease 2 | 80847014 | 79294414 | 98.08% | 12.127G | 10.826G | 89.27% | 97.91 | 93.73 | 47% | 69.80% |
| Disease 3 | 77508310 | 75432985 | 97.32% | 11.626G | 10.111G | 86.97% | 97.8 | 93.52 | 46% | 69.18% |
| Disease 4 | 79785044 | 77204174 | 96.77% | 11.968G | 10.462G | 87.42% | 97.82 | 93.64 | 48% | 71.14% |
| Disease 5 | 87214422 | 84815348 | 97.25% | 13.082G | 11.371G | 86.92% | 97.77 | 93.49 | 47% | 72.93% |
