# Supplementary material for: Genome-wide analysis of RNA-binding proteins co-expression with alternative splicing events in mitral valve prolapse
Source: Front Immunol. 2023 Apr 26;14:1078266. doi: 10.3389/fimmu.2023.1078266 (PMC10171460; doi:10.3389/fimmu.2023.1078266)
Supplement: Supplementary file 4 [file Table_3.docx]

| **Comparison of basic characteristics between MVP group and healthy group** | | | |
| --- | --- | --- | --- |
| Items | MVP group(n=5) | Healthy group(n=5) | p value |
| Age | 56.40±7.503 | 54.40±1.949 | 0.5799 |
| Male (%) | 3 (60%) | 3 (60%) | NA |
| Hypertension (%) | 3 (60%) | 3 (60%) | NA |
| Diabetes (%) | 0 (0%) | 0 (0%) | NA |
| Cerebral diseases (%) | 0 (0%) | 0 (0%) | NA |
| Respiratory diseases (%) | 0 (0%) | 0 (0%) | NA |
| Digestive diseases (%) | 1(20%) | 0 (0%) | >0.9999 |
| Renal diseases (%) | 0 (0%) | 0 (0%) | NA |
| Cancer history (%) | 0 (0%) | 0 (0%) | NA |

NA: Not accessible
